# The context of coping: a qualitative exploration of underlying inequalities that influence health services support for people living with long‐term conditions

**DOI:** 10.1111/1467-9566.12624

**Published:** 2017-10-11

**Authors:** Caroline M. Potter, Laura Kelly, Cheryl Hunter, Ray Fitzpatrick, Michele Peters

**Affiliations:** ^1^ Health Services Research Unit, Nuffield Department of Population Health University of Oxford Oxford UK; ^2^ Nuffield Department of Primary Care Health Sciences University of Oxford Oxford UK; ^3^ Department of Health England QORU: Quality and Outcomes of Person‐centred Care Policy Research Unit UK; ^4^ NIHR Collaboration for Leadership in Applied Health Research and Care (CLAHRC) Oxford Oxford UK

**Keywords:** long‐term conditions, chronic illness, coping, health services, social and cultural capital, qualitative interviews

## Abstract

Coping with chronic illness encapsulates both practical and emotional aspects of living life in relation to one's long‐term health condition(s). Dominant health psychology approaches for understanding coping, which underpin a more recent policy discourse on ‘self‐management’, focus sharply on the person affected by illness and potentially mask the influence of overarching social structure. In this paper we draw on qualitative interviews with 48 people living with long‐term conditions (LTCs), in order to highlight the role that structural configurations such as healthcare systems may play in either helping or hindering people's efforts to cope with chronic illness. We argue that coping is a social process in which health and related services, situated within their wider political‐economic contexts, play an active role in shaping people's attempts to live well with LTCs. More specifically, health systems are sites of social and cultural capital exchange that can differentially mobilise coping resources through access, continuity of care, and coordination across services. Whilst it is essential to recognise the personal agency of people living with chronic illness, it is also vital to acknowledge the underlying inequalities that affect the ways in which services can support such resourcefulness.

## Introduction

Long‐term conditions (LTCs) have emerged as the most pressing challenge facing healthcare systems today. In the context of aging populations and increasing multi‐morbidity (Barnett *et al*. 2012), the monitoring and management of chronic illness is a top priority in policy terms (WHO 2016). In England, people with long‐term conditions use a disproportionately high level of health and social care services, and the National Health Service (NHS) emphasises the prevention of LTCs and their risk factors as a key focus for action (NHS England 2014). The urgency surrounding this discourse suggests that, at present, the needs of people living with LTCs are not being fully met by health systems that have historically focused on single, acute conditions.

Coping with chronic illness encapsulates both practical and emotional aspects of living one's life in relation to one's health condition(s). Coping may be recognised along several dimensions, including the extent to which patients are able to follow and benefit from recommended treatment strategies (Leventhal *et al*. 1992), and the impact that LTCs have on their overall physical and emotional well‐being (Mosher *et al*. 2015). Within the literature coping has been extensively discussed, mainly within the field of health psychology (Folkman *et al*. 1986, Hagger and Orbell 2003, Leventhal *et al*. 1992, Yi‐Frazier *et al*. 2015). Much of this research has focused on the categorisation of various ‘coping strategies’ (Dunkelschetter *et al*. 1992, Dempster *et al*. 2015, Moskowitz *et al*. 2015, Silver *et al*. 2002), or alternatively on coping as a distinct process (Pennebaker *et al*. 1990) that overlaps with the concept of adaptation to illness (Charmaz 1995, Persson *et al*. 2013). Psychological studies on coping are supported by health‐related quality of life research that has led to the development of metrics such as the COPE inventory (Carver *et al*. 1989) and the Ways of Coping Questionnaire (Folkman and Lazarus 1988); these and other measures are used to assess aspects of coping (Kato 2015) and their relationships to health outcomes (Penley *et al*. 2002).

These dominant approaches for understanding coping focus almost exclusively on the individual affected by illness, for example their personal attributes (Taylor and Armor 1996) or their abilities to make behavioural / cognitive changes towards coping (Moskowitz *et al*. 2015). Even work on chronic illness that is sociologically informed (e.g. Charmaz 1995) tends to emphasise ‘identity work’ (e.g., reconciling one's notion of self to a changing body), with discussion of social influences limited to interpersonal relationships (Revenson *et al*. 1991) rather than overarching social structure. It is perhaps then unsurprising that this individualistic approach to chronic illness is becoming enshrined in policy language centred on a more specific notion of coping, namely ‘self‐management’ (Lorig 2010). Self‐management presupposes an ‘activated’ patient‐consumer who ‘co‐produces’ his/her health care through informed choice (Hibbard 2003). However as medical sociologists (e.g. Atkin *et al*. 2010, Moore *et al*. 2015, Ong *et al*. 2014) have noted, there are potential disconnects between self‐management as an instrumentalist policy goal and the meaningful practices that social actors might take in order to live well with LTCs.

Building on Atkin and colleagues' (2010: 392) conclusion that ‘coping is socially negotiated, defined by the social space in which it takes place’, in this paper we draw on qualitative interviews to highlight the role that structural configurations such as healthcare systems may play in either helping or hindering people's efforts to cope with LTCs. More specifically, we consider health and related services as sites for the generation and exchange of social and cultural capital (Bourdieu 1986, Sointu 2017) that can facilitate coping. Below we argue that coping is an ongoing social process, in which health systems (situated within their wider political‐economic contexts) actively shape the landscapes of resources that patients might mobilise when trying to cope with LTCs. Underlying society‐wide inequalities (i.e. differences in the amount and form of capital accrued by each person) can influence ways in which support from services is sought and delivered; only when these are acknowledged can system‐wide health inequalities be fully addressed.

## Methods

This qualitative study is part of a larger programme of research seeking to develop a new patient‐reported outcome measure, the Long‐Term Conditions Questionnaire (LTCQ) (Peters *et al*. 2016). Ethics approval was granted through the National Research Ethics Service (NRES) Committee London – Bromley (reference number: 14/LO/0834). The aim was to identify the issues and outcomes that matter most to people living with LTCs, which informed the content of the LTCQ. The analysis presented here is focused on how health and related services might enhance or inhibit a person's ability to cope with LTCs, reflecting two prominent themes that emerged from interviews.

### Recruitment of participants

Participants were recruited through eight primary care practices in Oxfordshire, or north‐west London, UK. Participants were invited to the study on the basis of having at least one of ten specified LTCs: cancer, chronic obstructive pulmonary disease (COPD), ischaemic heart disease (IHD), diabetes, depression, inflammatory bowel disease (IBD), multiple sclerosis (MS), osteoarthritis (OA), schizophrenia and stroke/transient ischaemic attack (TIA); these conditions were chosen to recruit a maximally diverse sample (Peters *et al*. 2016). Invited participants had been diagnosed more than 12 months previously so that they had adjusted to their diagnosis and had gained experience in living with their LTC(s). All participants were over 18 years old and able to communicate in English. Eligible patients were sent a letter of invitation by the primary care practice and were asked to contact the researchers if they wished to participate.

No participants with schizophrenia were recruited through primary care, so the data were supplemented with secondary analysis of interviews conducted previously with schizophrenia patients. These interviews stemmed from a study conducted in 2013/4 by RF and MP on outcomes valued by people with schizophrenia (Lloyd *et al*. 2017). Twenty‐two participants had been recruited through Rethink Mental Health, and six transcripts of diverse participants (in terms of age and gender) were selected for secondary analysis in this study. Ethics approval for the schizophrenia study had been gained through the East of Scotland Research Ethics Service (EoSRES) (Application Number 13/ES/0143).

### Interviews

Following initial contact that included confirmation of eligibility, the researchers arranged an interview time and location according to the preference of the participant; most interviews took place at the participant's home, or alternatively their place of work or the University. All participants gave written consent. The semi‐structured interviews were conducted between September 2014 and February 2015 by CH, LK and CP. All interviews were digitally audio‐recorded and transcribed verbatim by a professional transcription company.

The topic guide was informed by current literature on patient‐reported outcome measures, health care policy documents, three exploratory interviews with people with LTCs, and previous research with professional stakeholders (Hunter *et al*. 2015). Open‐ended interview questions focused on impacts on daily life and other outcomes of LTCs, personal strategies for looking after one's health in the context of LTCs, help needed or received in managing LTCs (including experiences of health, social care, or community/voluntary services), and goals or problems regarding LTC management. Interview times averaged 60 minutes.

### Analysis

Interviewers verified all transcripts before analysing the data in NVivo 10 (QSR International, Brisbane). Iterative framework analysis (Ritchie and Spencer 1994) was used; details of the development of the coding scheme have been described previously (Peters *et al*. 2016). The analysis presented here focused on two key themes that emerged as prominent across almost all interviews: ‘Sense of support from services’ (discussed in 47 out of 48 interviews) and ‘Coping with LTCs’ (discussed directly in 42 interviews). The parent theme on ‘Coping with LTCs’ included two sub‐themes: ‘coping through planning or adjusting way of living’ and ‘re‐prioritisation in light of LTC(s)’.

All raw data coded under ‘Coping with LTCs’ and its sub‐themes were read by the lead author and classified according to three broad types. Low‐level coping includes high emotional burden of illness (e.g. constant worry about the future), denial of the impact of illness, short‐term relief such as comfort eating, and/or self‐harm. Mid‐level coping includes recognition and basic acceptance of one's LTC(s), often reflected in adherence to recommended treatment. High‐level coping refers to an increased capacity for self‐awareness and self‐surveillance, which may be exhibited through proactive strategies for adjusting one's way of life in response to illness. These categories broadly correspond to Kato's (2015) meta‐analysis of relationships between coping strategies and outcomes, where high levels of distress (low‐level coping) were associated with emotional venting, denial, mental or behavioural disengagement, and disengagement through alcohol/drug use; while high levels of well‐being (high‐level coping) were associated with acceptance, planning, seeking social support, and positive reinterpretation of the situation.

All raw data coded under ‘Sense of support from services’ were also read by CP and were categorised as examples of participants either feeling supported or feeling unsupported, with broad descriptors for each. For example, some participants felt supported due to a trusting relationship with their primary care physician, access to specialist care, or knowledge of self‐management strategies gained through interactions with health professionals; in contrast, some participants felt unsupported due to impersonal (sometime inappropriate) care, lack of regular follow‐up with health professionals, or contradictions in approaches taken by multiple health professionals across disconnected services. Individual participants could have mixed experiences, that is, some examples of feeling supported and other examples of not feeling supported.

After all references to both the ‘coping’ and ‘support from services’ themes had been examined separately, individual summaries were written for each participant in order to explore potential relationships between support and coping. For interviews where no data had been coded under the ‘support’ and/or ‘coping’ themes (participants P04, P08, P17, P18, P27, PS6), CP referred back to the full interview transcripts and field notes; any further information that could be used to classify participants within these two domains (e.g. treatment compliance, indicative of mid‐level coping) was incorporated into the analysis. After summative analysis for each participant was complete, LK re‐read the raw coded data in order to verify each participant's classification. Following completion of the primary analysis, CP re‐read interview transcripts and fieldnotes to extract qualitative evidence of participants’ social and cultural capital, as these data were not systematically collected across interviews.

## Findings

A total of 48 interviews were included in the analysis, from 42 participants recruited through primary care and six through the schizophrenia study. Participants reported a wide range of LTCs in addition to those for which they were recruited. The age, gender, and LTCs reported by each participant are listed in Table 1.

**Table 1:** Participant characteristics, level of support and level(s) of coping

Participant	Gender, age	Long–term conditions	Level of support^a^	Level(s) of coping
P02	M, 69	cancer (lymphoma)	*somewhat unsupported* – well supported	high
P03	M, 71	type 2 diabetes, cancer (chronic lymphatic leukaemia)	somewhat unsupported	mid
P04	F, 80	osteoarthritis, hypothyroidism, hiatus hernia	*well supported* – somewhat supported	low, mid
P05	M, 88	diabetes, COPD, osteoarthritis, hypertension, gout, chronic liver disease	not supported	low, mid, high
P06	M, 87	cancer (Hodgkin's lymphoma), chronic skin condition	well supported	low, mid
P07	F, 54	multiple sclerosis	not supported	low, mid, high
P08	M, 69	stroke/TIA, epilepsy	well supported	mid
P09	F, 70	type 2 diabetes, hypothyroidism	mixed	high
P11	F, 44	depression, osteoarthritis	mixed	low, high
P12	F, 97	osteoarthritis, hypertension, hearing loss, chronic back pain, knee replacement, sciatica	well supported	mid
P13	M, 59	type 2 diabetes	mixed	low, high
P14	F, 76	depression, COPD, asthma, anxiety, osteoarthritis, diverticulitis	not supported	low, mid
P15	F, 66	multiple sclerosis	*not supported* – well supported	high
P16	M, 35	chronic renal failure, inflammatory bowel disease	*somewhat unsupported* – well supported	low, high
P17	F, 33	inflammatory bowel disease	mixed	low, mid, high
P18	F, 64	multiple sclerosis, polio complications, arthritis, stroke, vision problems	not supported	low, mid
P20	M, 75	ischaemic heart disease	somewhat supported	mid, high
P21	F, 58	diabetes, ischaemic heart disease, heart failure, chronic kidney disease	not supported	low
P22	M, 80	type 2 diabetes, ischaemic heart disease	mixed	mid, high
P23	F, 49	borderline personality disorder, type 2 diabetes, sciatica	not supported	low, mid
P24	M, 51	inflammatory bowel disease	not supported	low, mid, high
P25	M, 61	multiple sclerosis	*well supported* – somewhat unsupported	low, high
P26	M, 70	stroke, type 2 diabetes, cancer (non–Hodgkin's lymphoma), osteoarthritis, angina, hypertension, deep vein thrombosis (DVT)	*well supported* – mixed	low, high
P27	M, 65	type 2 diabetes, gout	mixed	mid
P28	F, 58	type 1 diabetes, asthma	not supported	low, mid
P29	F, 55	multiple sclerosis	not supported	low, high
P30	F, 68	type 2 diabetes, psoriatic arthritis	mixed	mid, high
P31	M, 77	ischaemic heart disease	mixed	mid
P32	M, 70	chronic back pain, type 2 diabetes, stroke, ischaemic heart disease, gout	mixed	mid, high
P33	M, 67	multiple sclerosis	somewhat supported	high
P34	M, 72	type 1 diabetes, osteoarthritis, asthma, polio–related complications	well supported	mid
P35	F, 64	COPD, stroke, ischaemic heart disease, osteoarthritis, agoraphobia, depression, atrial fibrillation, gout, spinal stenosis, deep vein thrombosis (DVT)	well supported	mid, high
P36	F, 65	multiple sclerosis	not supported	mid, high
P37	F, 66	inflammatory bowel disease, cancer (kidney – remission)	*somewhat unsupported* – mixed	mid, high
P38	M, 69	ischaemic heart disease	mixed	high
P39	F, 43	bipolar disorder	*not supported* – well supported	mid, high
P40	M, 31	depression, psychosis (drug–induced)	*not supported* – somewhat supported	mid, high
P41	M, 59	diabetes, osteoarthritis, circulatory problems	well supported	mid, high
P43	M, 59	neurofibromatosis type 1 (NF1), dyslexia	not supported	low
P44	F, 45	multiple sclerosis	well supported	low, mid
P45	F, 69	multiple sclerosis	mixed	low, mid
P47	M, 30	multiple sclerosis	somewhat unsupported	low, high
PS1	M, 45	paranoid schizophrenia, anxiety, depression, spinal stenosis	*not supported* – somewhat supported	low, mid
PS2	F, 29	paranoid schizophrenia, depression	not supported	low, mid
PS3	F, 58	paranoid schizophrenia, cancer (breast), hepatitis C	*mixed* – mixed	low, mid, high
PS4	M, 36	schizophrenia (paranoid psychosis)	*not supported* – supported	low, mid, high
PS5	M, 60	paranoid schizophrenia	well supported	mid
PS6	M, 30s	schizophrenia	somewhat unsupported	mid

a Levels of support in italics indicated *perceived levels of support in the past*, in comparison to current support levels (standard text)

Analysis of the data coded under ‘coping with LTCs’ revealed a striking finding: the heterogeneity of coping resources that could be utilised both within and across individual participants. Ability to cope with LTCs did not seem to follow a linear trajectory that the literature on adaptation to illness suggests (Chilton and Pires‐Yfantouda 2015). Rather, people living with LTCs could exhibit sophisticated strategies for self‐management and adjusting one's life in response to illness (classified as high‐level coping), while simultaneously referring to a high emotional burden of illness and/or behaviours such as comfort eating or substance abuse (classified as low‐level coping). Owing to the complexity of the concept, ‘coping’ could not be reduced to a single state. The majority of participants described at least two of the three levels of coping, as seen in Table 1.

In contrast, analysis of data coded under ‘sense of support from services’ suggested that individual participants could be classified at specific time points along a spectrum, from feeling unsupported to feeling well supported. Classification reflected each participant's overall confidence in services for addressing their health‐related needs, as conveyed at the time of interview. Participants at the ‘not supported’ end of the spectrum had little to no confidence that services would be there for them on a routine basis, or potentially even during times of crisis. Participants who felt generally unsupported recognised some ways in which services helped them, but they conveyed a lack of confidence in their quality, availability, or consistency overall. Some participants fell into a ‘mixed’ category, where they had confidence in some aspects of services but a lack of confidence in others; this applied to those who had received good support during acute bouts of illness but little follow‐up, where coordination across services was particularly fragmented (e.g. excellent support from specialists but poor support from primary care), or where participants had differing experiences of public versus private services. Those who felt generally supported conveyed an overall confidence in accessing and benefiting from services, but they highlighted specific instances of poorer care. Those at the ‘well supported’ end of the spectrum conveyed consistent benefit from services that were coordinated and in which participants held high trust.

Some participants reflected on their current sense of support from services in comparison to the past. Perceived support levels could shift over time in either direction. An upwards shift could reflect increased access to services or more effective treatment, for example after a correct diagnosis had been made. Alternatively, a decreased sense of support could reflect a more restricted range or quality of services in comparison to the past. Table 1 lists the sense of support from services that participants conveyed during interviews, with 11 of 48 participants indicating a change in their perceived support over time.

As indicated in Table 1, participants within each ‘sense of support’ grouping were able to draw on a range of coping resources. A more detailed exploration of particular cases shed light on suggested relationships between service support and coping. In the following sections, case comparisons are used to highlight three areas in which engagement with services might influence a person's ability to cope with LTCs: mutual acknowledgement of illness (enabled through access to services), continuity of relationships within medical settings, and coordination of action across the health system as a whole.

### Mutual acknowledgement of illness

Acknowledgement of one's illness is recognised as a fundamental aspect of coping (Folkman and Lazarus 1988); once a change in health status has been recognised, a range of resources can be mobilised to address its effects. Interview participants stressed the importance of recognising the impacts of LTCs on their lives, particularly in the context of medical encounters where such acknowledgement by health professionals can validate the legitimacy of patients' experiences. Access to services can create a social space where knowledge of illness is co‐constructed and coping resources are jointly mobilised by both structure (the health system) and agent (the person affected by the illness).

For some people, initiating a medical encounter to acknowledge illness is a difficult first step. P40, a 31‐year‐old man with a history of depression and drug‐induced psychosis, described a moment of crisis that led him to seek help. After many years of ‘being clever’ and ‘telling [doctors and counsellors] what they wanted to hear’ in response to his family's concerns about his mental health, as a young adult P40 was desperate to end the pain associated with his health conditions. He experienced ‘a blow’ when the first doctor he sought out responded to his attempted self‐harm with ‘Well, that's a bit stupid isn't it?’ His sense of being dismissed at this first moment of reaching out for help perpetuated the crisis, which reached its peak during a subsequent suicide attempt. Over time P40's engagement with health services improved, ultimately resulting in a diagnosis and follow‐up treatment. In acknowledging his illness with health professionals, he better understands and is able to identify resources for responding to it:

you know, all these psychiatrists and like people, like mental health people, they always talk about using tools to, you know, like cope with this and cope with that and, ‘I'm not going to see you for two weeks, what tools are you going to use?’ and for me it's just motivation … I can't let my daughter see what I used to be like and I'm not going to let my daughter grow up in a world where she can't go and talk to daddy for two days, because daddy's sitting in his room depressed, it's not going to happen.

Although P40 identified his main coping resource (his sense of responsibility to his family) as lying outside of health services, his engagement with medical professionals prompts such reflection and provides a space for ongoing understanding of his LTCs.

In contrast to the resources that can be activated through mutual acknowledgement of health conditions, delayed or incorrect diagnosis can engender feelings of distrust that might have longer‐term consequences for coping with LTCs. P07, a 54‐year‐old woman with multiple sclerosis, describes the anger that she felt at having not been correctly diagnosed before taking a major financial decision:

and he [senior consultant doctor] said ‘Oh, it'll probably … you've got something, probably never happen again’, and sent me away … we were just moving house and signing up for a massive mortgage, which I would not have done if I had known I had MS, but he didn't tell me … [later] my husband and I went along [to the primary care doctor for advice on fertility] and he said ‘You've got multiple sclerosis. Before you go ahead with having children you should know that you've got multiple sclerosis’. And I thought ‘Oh, my god, I've just signed this huge mortgage’.

P07 expresses worry that since her MS has never been monitored, she does not know how much her condition may have deteriorated over time. She describes a distant relationship with services, only requesting appointments for short‐term interventions such as an annual flu vaccine and never discussing her MS with a primary care doctor or practice nurse. Her sister, a health professional, has suggested that she pursue a baseline assessment and routine monitoring through private healthcare, which P07 thought was a good idea but which she had never considered before. Her initial experience of diagnosis and current distant relationship with services suggest a series of missed opportunities for services to have supported her more actively in recognising her health condition and in mitigating its impact on her life.

### Continuity of relationships

Beyond initial access to services and diagnosis, continuity of care can play a vital role in mobilising resources for coping with LTCs, through ongoing knowledge exchange and build‐up of trust with health professionals. However, many participants expressed dissatisfaction with inconsistencies of care. P14, a 76‐year‐old woman living with multiple LTCs including depression, COPD, and osteoarthritis, highlighted the frustration of presenting herself repeatedly to clinicians who had no prior knowledge of her health‐related history:

Now at the present one [primary care practice] it's not that there's anything wrong with it, but I've been there about five times and I've seen five different people … I mean they've got every right to differ [in medical opinion and advice given] but I find that very difficult. And if I have to you know, argue my case for anti‐depressants or sleeping tablets, I don't want to be seeing a different person and telling the same story you know, over and over.

For some participants, a lack of continuous care had led to broken relationships with services. P18, a 64‐year‐old woman, reflected on a lack of follow‐up after her stroke: ‘I just was left then to recover as best I could, and I did recover but my memory is still not brilliant’. P18 also experiences long‐term complications of polio and has been diagnosed with MS, for which she has similarly received no continuous care. She conveyed a hopeless attitude regarding the ability of services (including social services) to help her cope:

there's nobody willing to listen and help you with the things that you need the help with. So, you just get on with things the best you can and things you can't do you have to pay to have done for you … I had a stroke and again I thought maybe I'd get, at last, get some help, but you don't, it's a waste of time. So, I just … I don't bother asking any more, I just let it go.

In sharp contrast P39, a 43‐year‐old woman living with bipolar disorder, provides a powerful example of how continuity of care can positively influence coping. She contrasts her current situation to her previous 20‐year cycle of poor care, when early episodes of psychosis and self‐harm were dismissed by health professionals as something under her control. These earlier experiences led P39 to avoid medical help until symptoms became severe. In contrast, the care that she now receives enables her to address episodes of illness before they have serious consequences:

what's been different over the last 4 years, is because firstly I've got a care coordinator or a psychiatric nurse, I'm not actually sure of the title … I see her every 3 to 4 weeks. And that has made a huge difference, simply that they are keeping an eye on the whole purpose of not letting me get so manic and psychotic, or so suicidally depressed, that I actually then have to be admitted [followed by months or years of recovery] … the whole emphasis, like I say, has been on prevention, you know to ‘nip it in the bud’. As soon as they see symptoms, or things aren't happening, they react to it.

P39 goes on to describe the support provided during a recent illness episode by her care coordinator, who initiated a ‘bridge building’ service for liaising between P39's medical team and employer. The care coordinator facilitated a conference call in which P39 and her manager discussed the implications of her illness and agreed a phased strategy for returning to work. This continuous care not only worked to prevent joblessness, one of the major impacts of P39's long‐term condition, but also to increase her emotional well‐being; she expressed a sense of relief in no longer hiding her illness from her employer, and confidence that she will be supported through future experiences of illness. This case provides one of the most striking examples of how continuous care with health professionals can open up potential for shared treatment decisions and wider mobilisation of coping resources.

### System‐wide coordination

In parallel with the importance of continuity with trusted health professionals, participants raised a broader type of continuity that could affect coping resources: continuity at the structural level, across the health system as a whole. Even as some participants cited examples of excellent care at the individual level, many signalled disconnections of care such as long waiting times for appointments after referrals, or fragmented communication lines among health professionals working in different specialities.

Lack of coordination across services could particularly affect patients with multiple LTCs, who face an especially complex landscape to navigate when new symptoms or new health conditions emerge. P21, a 58‐year‐old South Asian woman, has a long history of diabetes and resultant chronic kidney disease; yet it is her more recently diagnosed heart problems that she most struggles to cope with. P21 describes her dismay at unsuccessful attempts to maintain registration with her hospital's cardiac department, and her resultant anxiety in making health decisions:

P21 You see I'm not worried about the diabetic problem, I'm not worried about my kidney problems, but I am worried for my heart problem because it's my every moment problem, and when I'm going up … I've got the toilet upstairs … I'm going by the stairs and then I have to wait [because of chest pains], and I'm just busting for the toilet you know, I just sit down [HEAVY SIGHS] … Sometimes there is a pain in the chest, there is a severe pain in the chest, and I'm just about to call the ambulance and then I'm thinking ‘if you're there with emergency people, you'll have to start from a, b, c, d’ because I'm not with the cardiac department now.

Interviewer So is it that you feel you don't want to have to explain everything to people, or that you don't feel you can explain to people?

P21 I can explain. To whom I should explain dear, to whom?

P21's current worries about when and how to seek treatment are set within her ongoing struggles for acknowledgement and support from services, particularly regarding her heart disease. Her initial report of chest pain was not taken seriously by her primary care doctor; P21 felt that this had led to delayed diagnoses and damage to her heart muscle, which in turn limited her options for treatment. She had sought support from her doctors in accessing both specialist cardiac care and social services (to address the issue of toilet accessibility at home) but had been denied both. She received conflicting advice from specialists who were not able to consider the impact of her health conditions as a whole; for example, she knew that exercise would potentially help her to manage her diabetes but felt unable to do it because of the pain and fatigue caused by her heart disease. Overall P21 was not coping well, conveying a high negative impact of her health conditions on her emotional well‐being.

Even among patients who are coping well, fragmentation of services can create a personal burden of coordination and knowledge‐sharing. P03, a 71‐year‐old man with type 2 diabetes and Chronic Lymphatic Leukaemia (CLL), expressed frustration that his primary care doctor (GP), who is effective at addressing his diabetes, knows so little about his CLL. P03 had discussed a persistent cough with his CLL specialist (accessed privately), who had given him a book about CLL that described the cough as a potential symptom. P03 had shared this information with his GP but felt that his GP still did not understand the issue. In the absence of an annual review to discuss the combined impacts of both conditions, P03 felt that neither of his doctors had a complete picture of his overall health: ‘this is your diabetes box, this is your CLL box, and never the twain shall meet’.

Beyond health care, a lack of coordination between health and social care services could also influence people's abilities to cope with LTCs. Within the UK these services are largely disconnected, funded by different government bodies that maintain distinct procedures for access. In the current political climate of fiscal austerity, some participants faced increasingly strict criteria for accessing some resources that had previously helped them to cope, even when their overall health status had not changed.

P26, a 70‐year‐old man, currently lives with a complex range of LTCs: diabetes, osteoarthritis, non‐Hodgkin's lymphoma, previous stroke, hypertension, angina, and deep vein thrombosis. He takes pride in the fact that he has never sought financial disability support from the state and conveys confidence in managing his medications. However he conveyed strong worry and anger about the recent denial of his disabled parking badge, which he had held for the previous six years. P26 highlighted the restricted activity that had resulted from this change: he is no longer able to do the shopping with his wife, take the dogs to the park, or join friends for dinner in town because he finds the extra distance that he would have to walk too taxing (breathlessness from his angina coupled with a heavy limp and restricted mobility from his osteoarthritis). At the time of interview this loss of a social care resource – which his doctor was in support of him having – was having the greatest negative impact on P26's life in relation to his LTCs.

The case examples presented above, reflecting the breadth of experiences of the wider sample, demonstrate that health and related services are not passive players in the lives of people with LTCs. People's ongoing abilities to cope with LTCs are set within the fluid levels of support that services may provide. Coping cannot be regarded as a purely individualistic pursuit of adaptation to illness. The wider contexts of people's lives, including their relationships with services, play an active role in enabling people to live well.

## Discussion: the hidden influence of social and cultural capital on services

This study demonstrates that coping with long‐term conditions is a dynamic process in which social institutions such as health services play an active role. While coping may appear to happen on a personal level, as an ongoing process coping emerges through people's interactions with their social and cultural environments. In the UK context, services face increasing financial constraints even as demand from an aging population continues to rise. Participants in our study were acutely aware of these pressures, conveying a sense of responsibility not to squander scarce resources. At the same time, some described instances where increasing restriction of resources had challenged or undermined their previous coping strategies. This study thus adds to sociological critiques of neoliberal approaches to health policy and medical practice, which can result in a moralising discourse that blames individuals for failures of non‐compliance or ineffective self‐management (Moore *et al*. 2015). Such hyper‐individualistic approaches mask the effects of political, economic and social processes on people's health, laying the groundwork for a systemic ‘structural violence’ (Farmer *et al*. 2006: 1686) that widens health inequalities.

The sample size in this qualitative study design necessarily limits the extent to which conclusions may be applied on a larger scale. These data do not allow for systematic comparisons of coping experiences across groups (e.g. along the lines of gender, ethnicity, and socioeconomic status), and we recognise the difficulty of attempting a structural analysis through detailed description of cases, where individuals' perceptions of services cannot be neatly distinguished from population‐level service organisation. However the breadth of experiences highlighted in the case examples indicate the complexity of coping as a process, with multiple factors (both personal and structural) coming in to play across time and space. Whilst it is essential to recognise the personal agency and resourcefulness of people living with chronic illness, even within health system structures that may be inadequate (Jowsey *et al*. 2016), we clearly cannot ignore the capacity of health services for enabling people to live well with LTCs. This study has highlighted examples of ways in which services can support such personal resourcefulness, and therefore facilitate coping, for example, through continuity of care and coordination across the health system.

At the same time, the heterogeneity of experiences presented by study participants within the supposedly homogenising context of a nationalised health service begs sociological explanation. Bourdieu's (1986) analysis of the forms of capital reminds us that knowledge and practice are generated at the nexus of structure and agency. People – drawing on their various forms of economic, social and cultural capital – encounter structures such as health and social care services that may enable or constrain them in their pursuit of health. From a sociological perspective services can thus be understood as sites of capital exchange, with some people relatively more enabled to pursue good health through these encounters than others.

Bourdieu argued that while economic capital was the most visible and immediately transferable form of capital, the importance of social and cultural capital for creating and maintaining ‘distinction’ among social groups cannot be ignored if we are to understand society‐wide inequalities, particularly those linked to class identity. In describing social capital he referred to the ongoing acts of exchange that give rise to senses of mutual recognition, obligation and reciprocity: the carefully chosen gift, words captured in letter, a marriage contract. These exchanges lead to ‘a durable network of more or less institutionalised relationships of mutual acquaintance and recognition’ (Bourdieu 1986:51) that can be called upon in times of need; social capital is membership of a group that allows one access to resources shared within that group. Cultural capital, in contrast, refers largely to embodied resources such as knowledge, skills, values, and preferences that each person acquires through a lifetime of social encounters, most notably during formative years of childhood development. Cultural capital may take objectivised forms (such as books or musical instruments) or institutionalised forms (such as educational attainments or professional qualifications), but these reflect their embodied forms. Cultural capital is on constant display through how one habitually thinks, speaks, and acts: durable dispositions of body and mind that Bourdieu (1984: 166) referred to as ‘habitus’.

While Bourdieu did not apply the forms of capital specifically to health, recent authors (Abel 2008, Pinxten and Lievens 2014, Sointu 2017) have argued for the relevance of his theory for explaining health disparities. Unequal access to economic capital is a necessary but insufficient explanation for observed variations in health experiences and outcomes. This observation particularly resonates in the context of publicly funded systems such as England's NHS, where the defining principle is the provision of safe and effective care in response to need, irrespective of ability to pay. In the cases presented above we demonstrated that in spite of the NHS's ethos of providing high‐quality care to all, people's relationships with services varied greatly, with a range of coping resources utilised at all levels of perceived support. We now suggest that variations in interactions with services reflect differences in the social and cultural capital that each actor brings to those encounters.

The possession of social capital facilitates initial access to services. Recall that P21 – a foreign‐born ethnic minority living on minimum wage – struggled to access the specialist health and social care services that she desperately required. The experiences of P38, a British white male professional, paint the opposite picture. After his referral to an NHS specialist was delayed:

at 8 o'clock on impulse, I phoned a cardiologist in [town] whom I knew well, [NAME], [CONNECTION WITH INTERVIEWEE'S WORK] and I said to him, ‘can I come and see you privately?’, and he said, ‘don't be daft, what's the problem?’, and I explained to him, and he said, ‘you better come and see me at 1 o'clock today’… and [after assessment] I think he thought I was going to drop dead on him, and he got me in that week, I went in on the Thursday, and had the operation on the Friday.

When standard care pathways did not meet his needs, P38 had other social resources to draw on. These cases are perhaps extreme counter‐examples, but they clearly illustrate how differences in social capital can translate to differential access to care; the contrasting relationships with services ultimately led to disparities in health outcomes.

Cultural capital is perhaps the least recognised resource that can explain variations in service support, which can in turn influence coping. Recent work by Sointu (2017) highlights the role of cultural capital in shaping healthcare interactions; in her qualitative longitudinal study with medical students, she demonstrated that commonalities of language and values led doctors to give preferential attention to ‘good’ patients who reinforce their competence while distancing themselves from ‘bad’ patients who challenge their abilities to help people. In our study, the importance of interacting with health professionals in their own terms was highlighted by P16, a young man who worked in medical communications and who had been diagnosed with chronic kidney disease as a teenager:

I'm 35, I'm technically very literate, so you know, computers and the internet don't phase me at all. I'm also very bossy and I take control of situations, and I'm very good at getting what I want in service situations … and sometimes I'm banging my head against a brick wall. Now what does a 75‐year‐old lady who doesn't speak English as a first language do, and how does she feel?

Beyond language, institutionalised cultural capital in the form of professional qualifications also helped some participants to interact more confidently with doctors, for example PS5 (a former GP) and P33 (a professional counsellor). Their experiences contrasted sharply to those of P43, who in addition to a chronic skin condition has dyslexia that prevents him from communicating with health professionals through expected written channels, and P23, who draws on state benefits and who felt ‘that I didn't have any control over what was going to happen to me, and that it was all in the hands of the doctors’.

These juxtaposed examples suggest that services can be understood as sites for exchanging capital in all their various forms (e.g. knowledge of illness), with some people better able to benefit from these interactions than others. While a range of coping resources was observed for participants at all perceived support levels, detailed case analysis indicates that greater capital enabled some people to thrive within medical encounters, or alternatively to draw on other resources to cope when they perceived service quality as poor.

## Conclusions

The results presented here move the concept of ‘coping’ well beyond its health psychology roots. Through a sociological lens coping must be understood not as an individual endeavour, but rather as an alignment of individual, health system, and societal resources within their fluid environments. Health and related services are sites of social and cultural capital exchange and are therefore important loci for mobilising coping resources through initial access, continuity of care, and system‐wide coordination. Coping with long‐term conditions thus does not happen merely at the level of individuals through pragmatic ‘self‐management’, nor is it limited to the intersubjective space between patients and their support networks. Coping emerges at the meeting point of personal agency and societal structures, where differences in social and cultural capital underpin inequalities that are deeply entrenched. In this paper we have brought to light some of the background difficulties that services face in trying to support all people with long‐term conditions equally well. Bourdieu's insights must not be lost if system‐wide health inequalities are to be fully addressed.
